# Ginger inhibits cell growth and modulates angiogenic factors in ovarian cancer cells

**DOI:** 10.1186/1472-6882-7-44

**Published:** 2007-12-20

**Authors:** Jennifer Rhode, Sarah Fogoros, Suzanna Zick, Heather Wahl, Kent A Griffith, Jennifer Huang, J Rebecca Liu

**Affiliations:** 1 88th Medical Group, Wright-Patterson AFB, Ohio, USA; 2 National Institutes of Health, National Human Genome Research Institute, Cancer Genetics Branch, USA; 3 Department of Family Medicine, University of Michigan, Ann Arbor, MI, USA; 4 Department of Obstetrics and Gynecology, Division of Gynecologic Oncology, University of Michigan, Ann Arbor, MI, USA; 5 Department of Biostatistics, University of Michigan, Ann Arbor, MI, USA

## Abstract

**Background:**

Ginger (*Zingiber officinale *Rosc) is a natural dietary component with antioxidant and anticarcinogenic properties. The ginger component [6]-gingerol has been shown to exert anti-inflammatory effects through mediation of NF-κB. NF-κB can be constitutively activated in epithelial ovarian cancer cells and may contribute towards increased transcription and translation of angiogenic factors. In the present study, we investigated the effect of ginger on tumor cell growth and modulation of angiogenic factors in ovarian cancer cells *in vitro*.

**Methods:**

The effect of ginger and the major ginger components on cell growth was determined in a panel of epithelial ovarian cancer cell lines. Activation of NF-κB and and production of VEGF and IL-8 was determined in the presence or absence of ginger.

**Results:**

Ginger treatment of cultured ovarian cancer cells induced profound growth inhibition in all cell lines tested. We found that *in vitro*, 6-shogaol is the most active of the individual ginger components tested. Ginger treatment resulted in inhibition of NF-kB activation as well as diminished secretion of VEGF and IL-8.

**Conclusion:**

Ginger inhibits growth and modulates secretion of angiogenic factors in ovarian cancer cells. The use of dietary agents such as ginger may have potential in the treatment and prevention of ovarian cancer.

## Background

In the United States, ovarian cancer is the most lethal gynecologic malignancy and represents the fifth leading cause of cancer death among women[1]. Key goals in the management of this disease are prevention, early detection, and prolongation of disease-free intervals and overall survival upon development of the disease. Most primary ovarian cancers arise from malignant transformation of the surface epithelium. Although the specific molecular events responsible for this transformation remain unknown, two general theories have been proposed: incessant ovulation [2,3] and excess gonadotropin secretion[4]. Ovulation is essentially a natural inflammatory process; therefore a pro-inflammatory state is felt contribute to ovarian carcinogenesis[5,6]. There is ample evidence that inflammation is causally linked to carcinogenesis [7] in other tumor types, and targeting mediators of inflammation has been used as a strategy to both prevent and treat cancer.

Our understanding of ovarian cancer carcinogenesis is limited. Many of the genes that mediate inflammation and adaptive survival strategies in cancer cells including: self-sufficient growth, insensitivity to growth-inhibitory signals, evasion of apoptosis, limitless replicative potential, and sustained angiogenesis,[8] are under the transcriptional control of NF-κB [9]. Constitutive activation of NF-κB has been described in many tumor types including ovarian cancer [9], suggesting that targeting NF-κB may have anti-inflammatory and anti-neoplastic effects in this tumor type.

Of late, several plant-derived extracts have been evaluated as possible inhibitors of the NF-κB pathway. Ginger root (*Zingiber officinale *radix Roscoe) and its main poly-phenolic constituents (gingerols and zerumbone) have anti-oxidant [10-15], anti-inflammatory [16-19], and anti-carcinogenic activity [20-24]. In particular, ginger root and its constituents can inhibit NF-κB activation induced by a variety of agents [25-28], and has been shown to down regulate NF-κB regulated gene products involved in cellular proliferation and angiogenesis, including IL-8 [29], and VEGF[30]. These factors have also been shown to promote tumor cell proliferation, angiogenesis, and affect apoptotic response in ovarian cancers.

Among the myriad of pro-angiogenic cytokines known to induce tumor angiogenesis, vascular endothelial growth factor (VEGF) is the best characterized. *In vitro *and *in vivo *studies have shown that VEGF is critically involved in various steps of ovarian cancer carcinogenesis, and recent studies indicate that serum VEGF is an independent prognostic factor for patients with all stages of ovarian cancer [31]. Interleukin-8 (IL-8) was originally found to function as a macrophage derived pro-angiogenic factor [32], and has since been shown to affect cancer progression through mitogenic, angiogenic and motogenic effects[33]. Increased blood levels of IL-8 have been found in ovarian cancer patients [34], and IL-8 has been shown to stimulate proliferative growth in ovarian cancer cells *in vitro*[35].

In the present study, we tested the hypothesis that ginger could exert inhibitory effects on cell growth, and modulate the production of angiogenic factors in epithelial ovarian cancer cells. Our data reveals that ginger significantly inhibits ovarian cancer cell growth, and that the major bio-active component of ginger is 6-shagoal. Moreover, ginger inhibits NF-κB activation and subsequent secretion of the angiogenic factors IL-8 and VEGF in ovarian cancer cells.

## Methods

### Chemicals

Dried whole ginger root powder extract (1:1 extraction solvent: ethanol 50 percent/water 50 percent {}) standardized to 5% gingerols, was obtained from Pure Encapsulations, Inc (Sudbury, MA.). All studies were conducted using a single batch of ginger root extract. Content of gingerols in the ginger root extract were independently verified using appropriate high performance liquid chromatography methods [36]. The total gingerol content in the ginger root extract (12.3 mg/250 mg (4.9 percent) was confirmed the end of the study at Integrated Biomolocule (Tuscon, AZ). For *in vitro *studies, a stock solution was prepared by vortexing 50 mg of powder into 1 ml of aqueous dimethyl sulfoxide (DMSO). Insoluble particulates were centrifuged to the bottom of the eppendorf tube and the supernatant was then further diluted into cell culture media in the concentrations described. Cisplatin was obtained from Bedford Laboratories (Bedford, OH.) Ginger standards 6-gingerol, 8-gingerol, 10-gingerol and 6-shogaol were purchased from ChromaDex (Santa Ana, CA.) Standards were solubilized in DMSO and molarity was determined per supplier recommendations. Sulforhodamine B was obtained from Sigma-Aldrich, Inc. (St. Louis, MO.)

### In Vitro Growth Inhibition Assays

The Sulfhodamine B assay was used according to the method of Skehan *et al.*[37]. Cells were plated in a 96 well format (3 × 10^3 ^cells/well) and twenty-four hours after plating, DMSO, ginger, or ginger component standards for indicated time periods. At the end of drug exposure, cells were fixed with 50% trichloroacetic acid and stained with 0.4% sulforhodamine B (Sigma-Aldrich, St. Louis, MO), dissolved in 1% acetic aid (100 μl/well) for 30 minutes, and subsequently washed with 1% acetic acid. Protein-bound stain was solubilized with 150 μl of 10 mM unbuffered Tris base, and cell density was determined using a colorimetric plate reader (wavelength 570 nm). All samples were run in triplicate. Cell number and viability of treated cells were confirmed using the trypan blue dye exclusion assay.

### Cell Lines, Plasmids and Immunoblotting

SKOV3 ovarian cancer cells were obtained from the American Type Culture Collection (Manassas, VA.) Dr. K. Cho (University of Michigan) generously provided A2780, CaOV3, and ES2 cell lines. SKOV3, CaOV3, and ES-2 cells were originally harvested from patients with recurrent ovarian cancer. Ovarian cancer cells were maintained in DMEM supplemented with 10% fetal bovine serum, 100 units/ml penicillin and 100 mg/ml streptomycin (Invitrogen Corporation, Grand Island, NY.) Human ovarian surface epithelial cells were obtained, after Institutional Review Board approval, from patients undergoing surgery for non-ovarian cancer gynecologic indication. Cells were initially cultured in Medium 199/105 (1:1) supplemented with 10% fetal bovine serum, 100 units/ml penicillin and 100 mg/ml streptomycin and EGF 10 ng/ml during primary culture. After establishing adequate growth, cells were cultured with the above media, excluding EGF, prior to use in assays[38]. CaOV3 and SKOV3 cell lines were transfected with the indicated expression plasmid using LipofectAMINE Plus, or AMAXA electroporation respectively.

### NF-κB promoter-dependant Luciferase Reporter Gene Activation

CaOV3 and SKOV3 cells were plated in 12 well plates. Twenty-four hours after plating, cells were transfected the reporter plasmid pBVIx-Luc. This plasmid contains six NF-κB recognition sites within the promoter sequence linked to the luciferase reporter gene, and was generously provided by Dr. Valerie Castle (University of Michigan, Ann Arbor, MI). Following transfection, cells cultured overnight, then treated with DMSO vehicle control or ginger (75 μg/ml). Following incubation with ginger for 6 hours, cells were harvested, and luciferase activity was determined using a Monolight 2010 luminometer.

### VEGF and IL-8 ELISA

Production of VEGF and IL-8 was determined in A2780, CaOV3, ES2, and SKOV3 cells. IL-8 concentrations were undetectable (<0.05 pg/ml) in A2780 and CaOV3 cells lines (data not shown). Cells were cultured in a 96 well format overnight, and then treated with DMSO vehicle control or ginger (75 μg/ml). After 48 hours, cell supernatant was removed and assayed using a commercial ELISA kit from R&D Systems (Minneapolis, MN). Assays were performed in triplicate and concentrations of VEGF and IL-8 (pg/ml) were compared with standard curves obtained with human recombinant VEGF_165 _and IL-8 provided with the kit.

### Statistical analysis

Standard analysis of variance techniques were used to compare between cell types, or culture conditions, depending on the analysis of interest. An overall F-test was used to determine if there was at least one significant difference between the groups tested. Tukey's honestly significantly different (HSD) multiple comparison procedure was used to determine significant pairwise comparisons, while assure the overall type I error rate was 5% or less. When the comparisons of interest were between treatments and the control condition alone, Dunnett's multiple comparisons technique for a single control was used.

## Results

### Ginger inhibits growth in ovarian cancer cells as compared to non-transformed ovarian epithelial cells

**Figure 1:**
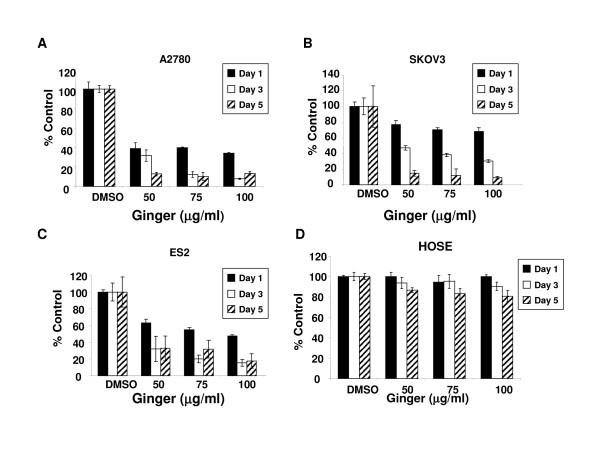
**Continuous ginger exposure inhibits growth in ovarian cancer cells *in vitro***. A, B, C: The effect of ginger on growth of A2780, SKOV3 and ES2 ovarian cancer cell lines was assessed by using sulforhodamine B assays. Cells were incubated continuously with media containing ginger at the indicated concentrations and growth was assayed at Days 1, 3 and 5 of exposure. Ginger treated cells displayed significant growth inhibition as compared to control treated cells (p < .05 for all cell lines, all ginger concentrations). D: Human ovarian surface epithelial cells were treated with the indicated concentrations of ginger with minimal effect seen following days 1–3 of culture (p > .05). HOSE demonstrated some inhibition of growth by day 5 of treatment (p < .05). Data are presented as means ± S.D, and are representative of at least 3 independent experiments.

**Figure 2:**
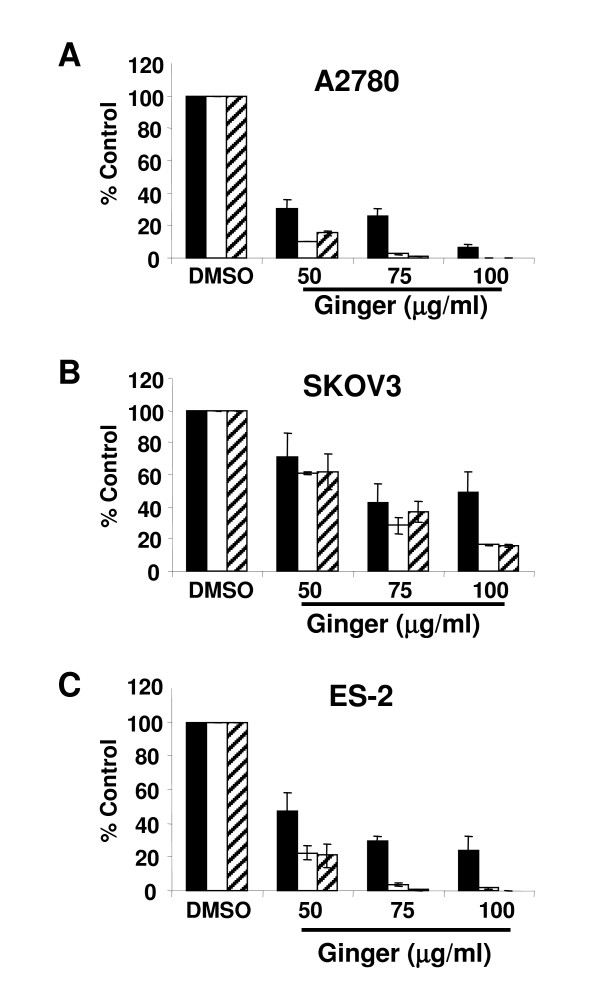
**Continuous ginger exposure inhibits growth in ovarian cancer cells *in vitro***. A, B, C: The effect of ginger on growth of A2780, SKOV3 and ES2 ovarian cancer cell lines was assessed using the trypan blue exclusion assay. Cells were incubated continuously with media containing ginger at the indicated concentrations and viable cells were counted on days Days 1, 3 and 5 of exposure. Ginger treated cells displayed significant growth inhibition as compared to control treated cells.

Continuous exposure to ginger extract resulted in a marked reduction in cell growth after 1–5 days of exposure in A2780 ovarian cancer cells (Figure 1A, p < .0001 at all doses and time points). We tested additional ovarian cancer cell lines to determine if this was an effect unique to the A2780 ovarian cancer cells. Ginger treatment resulted in similar effects in all cell lines tested, including the chemoresistant cell lines SKOV3 and ES-2[39] (Figure 1B,C, p < .05 for all doses and time points). Untransformed human ovarian surface epithelial cells (HOSE) were minimally affected by ginger extract exposure at days 1 and 3, and showed some inhibition in growth by day 5 (Figure 1D, p > .05 for days 1 and 3, p < .05 for day 5). To confirm that ginger treatment inhibited cell growth, treated cells were analyzed by trypan blue exclusion as well. As expected, ginger treatment resulted in a profound inhibition of cell proliferation and growth at doses of 50 μg/ul and higher (Figure 2).

**Figure 3:**
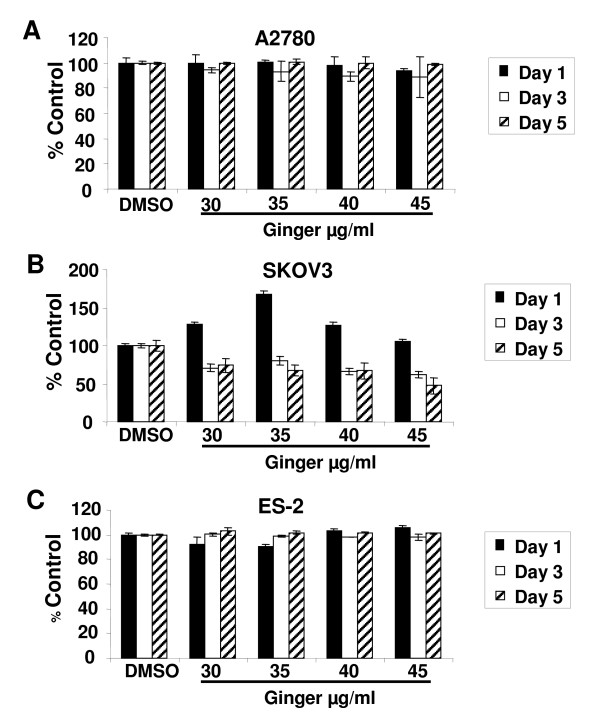
**Low concentrations of ginger have minimal effect on ovarian cancer cell growth**. A, B, C: The effect of ginger on growth of A2780, SKOV3 and ES2 ovarian cancer cell lines was assessed by using sulforhodamine B assays. Cells were incubated continuously with media containing ginger at the indicated concentrations and growth was assayed at Days 1, 3 and 5 of exposure. Using these concentrations of ginger, only SKOV3 cells displayed diminished cell growth.

To determine whether lower doses of ginger could also inhibit cell growth, an extended range of concentrations was tested. In the A2780 and ES-2 cell lines, ginger concentrations of less than 50 μg/ml did not significantly impact cell growth, whereas in the SKOV3 cell line, some inhibition of cell growth was seen with ginger concentrations as low as 30 μg/ml (Figure 3 and data not shown).

### 6-Shogaol is the most active of the individual ginger components tested in ovarian cancer cells

**Figure 4:**
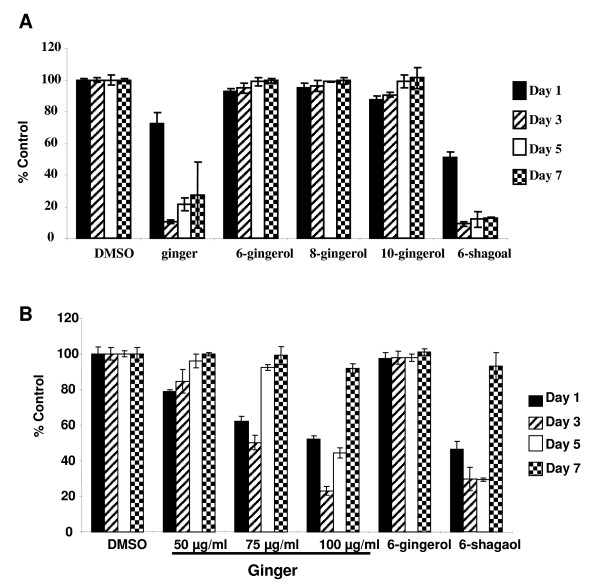
**6-shogaol is the most effective individual ginger component in ovarian cancer cells. Transient ginger exposure results in a non-sustained decrease in proliferation of ovarian cancer cells**. A: A2780 cells were treated for 24 hours with 75 μg/ml of ginger extract or 7.5 mM of each of the ginger standards. Media containing the indicated compounds was replenished at day 3. Growth was assayed via sulforhodamine B assays on Days 1, 3, 5, and 7. Data are presented as means ± S.D. B. A2780 cells were treated for 24 hours with indicated concentrations of ginger extract or 7.5 mM of 6-gingerol and 6-shogaol. Media containing the indicated compounds was washed off and replaced with complete media after 24 hours. Growth was assayed via sulforhodamine B assays on Days 1, 3, 5, and 7. Data are presented as means ± S.D.

**Figure 5:**
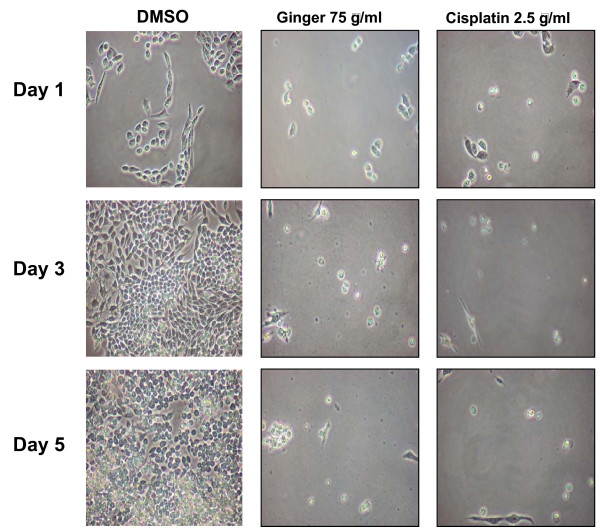
**Morphologic appearance of ginger treated ovarian cancer cells**. A2780 ovarian cancer cells were incubated with DMSO solvent, ginger (75 μg/ml, replenished on day 3), or Cisplatin (2.5 μg/ml). Cells were examined by light microscopy at 1, 3, and 5 days of treatment.

Previous investigators have shown bio-activity of various individual ginger components in several tumor types [30,40-43]. To determine the relative bio-activity in ovarian cancer, A2780 ovarian cancer cells were treated with 6-, 8- and 10-gingerol as well as 6-shogaol. In contrast to other published findings, we found that 6-, 8- and 10-gingerol had no effect on the growth or viability of ovarian cancer cells (p > .05 at all time points). Treating cells with whole ginger extract or 6-shogaol resulted in profound growth inhibition (Figure 4A, p < .05 at all time points for both ginger and 6-shogaol treated cells). Morphologically, cells treated with ginger appeared markedly growth inhibited, similar to cisplatin treated cells (Figure 5). Cells cultured with vehicle control (DMSO) continued to proliferate.

We next determined if continuous exposure to individual ginger components was necessary to cause the growth inhibitory effect seen in ovarian cancer cells. Similar to the use of whole ginger root extract, continuous exposure to 6-shogaol was necessary to cause the growth inhibitory effect seen in ovarian cancer cells. We treated cells with ginger and individual ginger components for 24 hours, after which the cells were washed and media was changed. Once ginger or 6-shogaol was removed from the media, cell growth resumed (Figure 4B).

### Ginger inhibits NF-κB in ovarian cancer cells

**Figure 6:**
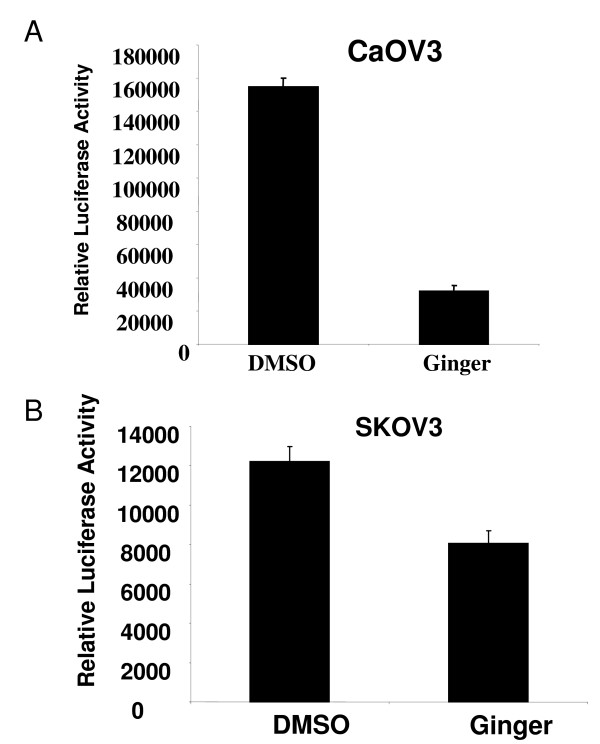
**Ginger inhibits NF-kB activation in ovarian cancer cells**. Representative ovarian cancer cell lines with high endogenous NF-kB activation, (A) CaOV3 and (B) SKOV3 were transfected with an NF-κB-dependent reporter plasmid (pBVIx-Luc). Cells were treated with DMSO (vehicle control) or ginger (75 μg/ml). NF-κB activation was determined by measuring relative luciferase activity 48 hours after treatment. Luciferase activity is reported as arbitrary relative light units (mean +/- S.D.) Ginger treatment resulted in inhibition of NF-κB activation (p < .05 for both cell lines). Representative data is shown.

Because we found that ginger markedly suppressed ovarian cancer cell proliferation *in vitro*, and several genes that regulate proliferation are regulated by NF-κB, we hypothesized that ginger may mediate its anti-neoplastic activity in ovarian cancer cells though modulation of this pathway. Constitutive activation of NF-κB has been described in many tumor types including ovarian cancer[9], suggesting that targeting NF-κB may have an anti-neoplastic effect in this tumor type. Natural products such as ginger, or ginger components such as zerumbone can inhibit NF-κB in other cell types [16,44,45]. We chose two chemoresistant ovarian cancer cell lines (CaOV3 and SKOV3) to evaluate the effect of ginger treatment on activation of NF-κB. As shown in Figure 6, treatment with ginger extract resulted in a significant inhibition of NF-κB activation in CaOV3 and SKOV3 cell lines

### Ginger Inhibits IL-8 and VEGF Secretion in Ovarian Cancer Cells

**Figure 7:**
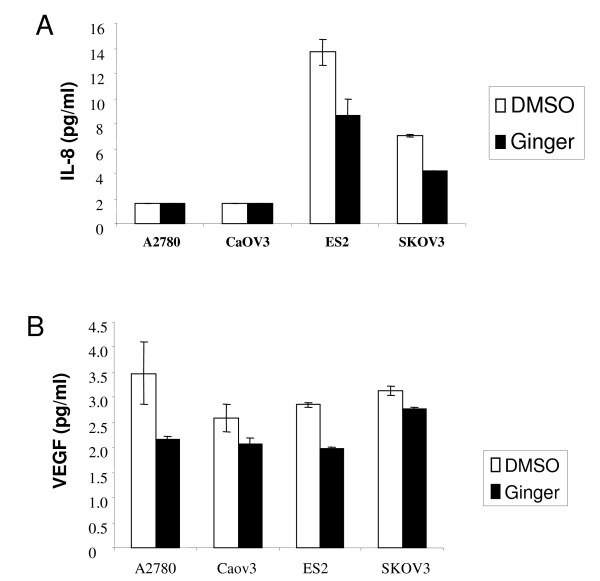
**Ginger inhibits VEGF and IL-8 in ovarian cancer cells**. Ovarian cancer cells were cultured with DMSO (vehicle control) or Ginger (75 μg/ml) for 48 hours. Production of the angiogenic factor s IL-8 (A) and VEGF (B) were assayed using ELISA assays. (A.) Only ES-2 and SKOV3 cells expressed high IL-8 levels at Baseline, and ginger treatment resulted in a significant decrease in IL-8 production (p < .05 for both cell lines). (B.) VEGF production was reduced in all cell lines following ginger treatment (p = .19, .18, .007, and .07 for A2780, CaOV3, ES-2 and SKOV3 cells respectively).

IL-8 can function as a paracrine and/or autocrine growth factor in some tumor types, and the secretion of IL-8 protein from tumor cells themselves is thought to be crucial for these effects[46,47]. In ovarian cancer patients, elevated IL-8 expression has been found in ascites as well as in serum[33]. Furthermore, IL-8 has been shown to stimulate proliferative growth in ovarian cancer cells *in vitro*[35]. Because IL-8 secretion is thought to be regulated in part by NF-κB, and ginger can clearly inhibit NF-κB in ovarian cancer cells, we hypothesized that ginger could also inhibit IL-8 secretion. Using a representative panel of ovarian cancer cell lines, we found that A2780 and CaOV3 cells produced negligible amounts of IL-8 (<0.05 pg/ml), whereas the cell lines ES-2 and SKOV3 had high constitutive expression of IL-8 (Figure 7A). Treatment with ginger resulted in significant inhibition of IL-8 production in the ES-2 and SKOV3 cell lines (p < .05 for both cell lines).

VEGF, the most important inducer of angiogenesis, is also under transcriptional control of NF-κB[9]. Serum VEGF levels as well as tumor expression of VEGF are associated with poor prognosis in ovarian cancer patients [31], and inhibition of VEGF function using Avastin™ has shown promise in the treatment of ovarian cancer patients[48]. Because ginger treatment resulted in inhibition of NF-κB, we next sought to determine whether ginger could similarly inhibit VEGF in ovarian cancer cells. In all cell lines tested, there was high endogenous production of VEGF, and ginger treatment resulted in inhibition of VEGF secretion. Inhibition of VEGF secretion was most evident in the ES-2 cell line (p = .007), as compared to the other cell lines tested (p = .19, .18, and .07 for A2780, CaOV3, and SKOV3 respectively, Figure 7B).

## Discussion

The analysis of epidemiologic data and disparities in global incidence of ovarian cancer may provide clues to uncover environmental and biologic factors that contribute towards the development of ovarian cancer. Dietary prevalence of foods such as ginger, garlic, soy, curcumin, chilies and green tea are thought to contribute to the decreased incidence of colon, gastrointestinal, prostate, breast and other cancers in South East Asian countries [49]. Accumulating evidence suggests that many dietary factors may be used alone or in combination with traditional chemotherapeutic agents to prevent or treat cancer. The potential advantage of many natural or dietary compounds seems to focus on their potent anticancer activity combined with low toxicity and very few adverse side effects.

Epithelial ovarian carcinoma is the leading cause of death among patients with gynecologic cancers. Despite multiple modalities of treatment including surgery and chemotherapy, ovarian cancer patients continue to have one of the lowest 5-year survival rates [1]. The significant morbidity and limited success of surgery and chemotherapy for ovarian cancer has led to searches for alternative therapies. Recently, ginger root and its main poly-phenolic constituents (gingerols and zerumbone) been shown to exhibit anti-inflammatory [16-19], and anti-neoplastic activity [20-24] in several cell types through inhibition of the transcription factor NF-κB [25-28]. NF-κB plays an important role in tumorigenesis, given its ability to control the expression and function of numerous genes involved in cell proliferation, sustained angiogenesis, and evasion of apoptosis. Different tumor types, including ovarian cancer, have been shown to express high constitutive NF-κB activity[9]. In this study we show that ginger blocks NF-κB activation in ovarian cancer cells, resulting in inhibition of NF-κB regulated gene products involved in cellular proliferation and angiogenesis.

Many of the pathways that mediate adaptive survival strategies in cancer cells are under the transcriptional control of NF-κB [9]. We have shown here that in ovarian cancer cells, NF-κB is constitutively activated, and blocking NF-κB activation with ginger results in suppressed production of NF κB regulated angiogenic factors and selectively inhibits ovarian cancer cell growth. We have found that ginger selectively inhibits ovarian cancer cell growth, as compared to non-transformed ovarian epithelial cells. Previous reports indicate that the ginger component 6-shogaol induces cell death in chemoresistant hepatoma cells [50], yet inhibits cell death in non-neoplastic spinal cord cells [51], suggesting that ginger and ginger components' effects are cell type specific. The apparent contradictory findings may be due to a differential effect of ginger on transformed cells (i.e. cancer cells) vs. untransformed cells. Phytochemicals such as ginger, generally have multiple molecular targets. This pleiotropism may constitute an advantage in the treatment of ovarian cancer, where multiple factors contribute towards the carcinogenic process.

## Conclusion

The results of this study indicate that ginger may exhibit anti-neoplastic effects through the inhibition of NF-κB. Further studies utilizing ginger in an *in vivo *model of ovarian cancer will provide a platform for the development of ginger as a therapeutic tool in this disease.

## Abbreviations

NF-κB (nuclear factor-kappa B), VEGF(vascular endothelial growth factor), IL-8 (Interleukin-8), HOSE (human ovarian surface epithelial cells)

## Competing interests

The author(s) declare that they have no competing interests.

## Authors' contributions

SZ and JRL developed the study design. SZ provided whole ginger root powder and coordinated verification of gingerol content in the ginger root extract. JR, SF, HW, and JH performed the experiments. JRL coordinated design and interpretation of experiments.

## Pre-publication history

The pre-publication history for this paper can be accessed here:


